# A New Method to Improve Running Economy and Maximal Aerobic Power in Athletes: Endurance Training With Periodic Carbon Monoxide Inhalation

**DOI:** 10.3389/fphys.2019.00701

**Published:** 2019-06-06

**Authors:** Jun Wang, Yunhui Ji, Li Zhou, Yang Xiang, Ilkka Heinonen, Peng Zhang

**Affiliations:** ^1^The Belt and Road Joint Laboratory for Winter Sports, Department of Exercise Physiology, Beijing Sport University, Beijing, China; ^2^Department of Physical Education, Shanxi Medical University, Taiyuan, China; ^3^School of Physical Education, Yan’an University, Yan’an, China; ^4^Turku PET Centre, Department of Clinical Physiology and Nuclear Medicine, University of Turku, Turku, Finland; ^5^Rydberg Laboratory for Applied Sciences, Department of Environmental and Biosciences, Halmstad University, Halmstad, Sweden; ^6^Department of Exercise Science, East Stroudsburg University of Pennsylvania, East Stroudsburg, PA, United States

**Keywords:** carbon monoxide, endurance training, EPO, total hemoglobin mass, running economy, maximal aerobic power

## Abstract

**Background:**

Altitude training stimulates erythropoietin hormone (EPO) release and increases blood hemoglobin (Hb) mass, which may result in improved oxygen (O_2_) transport capacity. It was hypothesized in the present study that periodic inhalation of carbon monoxide (CO) might elicit similar physiological adaptations compared to altitude training.

**Methods:**

Twelve male college student athletes, who were well-trained soccer players, participated. They performed a 4-week treadmill-training program, five times a week. Participants were randomly assigned into an experimental group with inhaling CO (INCO) (1 mL/kg body weight for 2 min) in O_2_ (4 L) before all training sessions and a control group without inhaling CO (NOCO). CO and EPO concentrations in venous blood were first measured acutely at the 1st, 2nd, 4th, 6th, and 8th hour after INCO, and total hemoglobin mass (tHb), running economy and VO_2_max were measured before and after the 4 weeks training intervention.

**Results:**

HbCO% increased from 0.7 to 4.4% (*P* < 0.05) after 1 h of CO inhalation and EPO increased from 1.9 to 2.7 mIU/mL after 4 h post CO inhalation (*P* < 0.05) acutely before the intervention. After the training, the tHb and VO_2_max in the INCO group increased significantly by 3.7 and 2.7%, respectively, while no significant differences were observed in the NOCO condition. O_2_ uptake at given submaximal speeds declined by approximately 4% in the INCO group.

**Conclusion:**

Acutely, EPO increased sharply post CO inhalation, peaking at 4 h post inhalation. 4-weeks of training with CO inhalation before exercise sessions improved tHb and VO_2_max as well as running economy, suggesting that moderate CO inhalation could be a new method to improve the endurance performance in athletes.

## Introduction

Altitude training has been widely applied in endurance sports for enhancing athletes’ maximal aerobic power (Levine and Stray-Gundersen, 1997). The main physiological mechanism of the maximal aerobic power improvement may result from altitude training stimulating the secretion of EPO from the kidneys, which increases the formation of red blood cells (RBC) from the bone marrow and raises circulating total hemoglobin (tHb) mass (Chapman et al., 1998). Consequently, altitude training increases the ability of an individual to transport O_2_ to the working muscles, which enhance exercise power. In addition, altitude training has been documented to improve performance through enhancing the ability of skeletal muscle to buffer lactate and improving running economy (Gore et al., 2001; Saunders et al., 2004).

Carbon monoxide (CO) is an important endogenous molecule that can also be inhaled (Prabhakar, 1998). It has long been established that CO has an Hb binding ability greater than that of O_2_. Therefore, when an individual inhales CO, this can be considered to induce mild hypoxia *in vivo*, as carboxy-hemoglobin (HbCO) is incapable of carrying enough O_2_ and there is reduced amount of O_2_ bound to Hb and delivery to active muscle. As persistent CO inhalation reduces Hb ability to carry O_2_, acute exposure to small doses of CO could, in theory, induce similar performance benefits to that of altitude training. Previous research showed that increased ventilation in mild exercise rapidly removes CO from the blood in humans (Zavorsky et al., 2012). The clearance half-time in males is slower in comparison to their female counterparts due to their larger tHb (Zavorsky et al., 2014). If CO can enhance sporting performance, a sample of well-trained male individuals, who can have up to a 37% higher tHb in comparison to untrained athletes, (Kjellberg et al., 1949) is a suitable subject population as CO can remain in the blood for prolonged periods of time. Hence, it is hypothesized that an appropriate dosage of inhaled CO might have sufficient time to provoke mild hypoxia before clearance, causing similar physiological adaptive responses to that of altitude training.

Since a periodic inhalation of small amounts of CO at sea level might have potential to simulate the hypoxic effects of altitude and increase tHb mass and enhance maximal aerobic power, it is worthwhile to investigate if CO inhalation could offer a more convenient and less expensive method of improving exercise performance. In a study, by Fröscher and Uhlmann (2016) 10 days of intermittent, low-dose CO inhalation at rest did not lead to improvements in Hb mass or aerobic peak power, but to the best of our knowledge combined inhalation of CO and exercise training intervention has not been performed. Consequently, the aim of this study was to examine the effect of inhaling a small amount of CO on EPO secretion and total hemoglobin mass (tHb), running economy and maximal aerobic power in a combination with treadmill training in well trained young adults.

## Materials and Methods

### Subjects

Twelve healthy young adult soccer players from Beijing Sport University were recruited in this study by network and lecture recruitment. All subjects were living at low altitude, were non-smokers and had never participated in altitude training or hypoxic research, while they had participated in sport training for more than 10 years prior to the experiments. Moreover, all subjects understood the protocol and risks of the experiment and provided written informed consent. The study was approved by the University’s Sports Science Experimental Ethics Committee. A description of the subjects is presented in Table 1.

**Table 1:** Anthropometric data and pre-training VO_2_max.

Group	Age (years)	Height (cm)	Weight (kg)	BMI (kg/m^2^)	Training (years)	VO_2_max (mL/kg/min)
INCO (*n* = 6)	20 ± 0.3	180 ± 4.8	70 ± 3.5	22 ± 1.5	10 ± 0.2	58.3 ± 2.3
NOCO (*n* = 6)	21 ± 0.8	180 ± 1.6	71 ± 3.6	22 ± 0.9	10 ± 0.4	58.4 ± 7.4

*Data is presented as means and standard deviations. INCO = training group with CO inhalation; NOCO = control group with no CO inhalation. There were no significant differences between groups in any variable.*

### Test Protocol

#### One-Time CO Inhalation Testing

**FIGURE 1:**
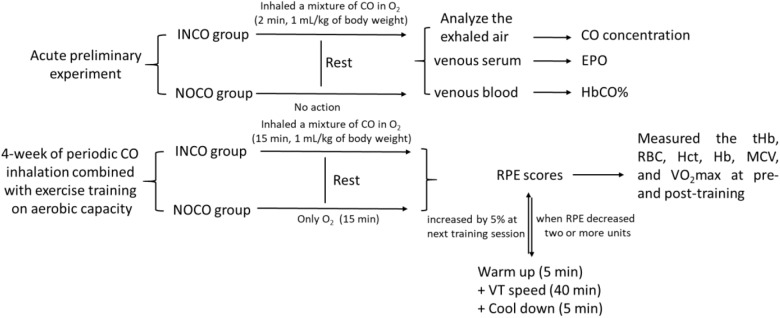
The test protocol of study. The INCO, NOCO, VT, tHb, RBC, Hct, Hb, MCV, and RPE are training group with CO inhalation, control group with no CO inhalation, ventilatory threshold, total hemoglobin, red blood cells, hematocrit, hemoglobin, mean corpuscular volume, and ratings of perceived exertion, respectively.

The study was divided into two separate experimental sections (Figure 1). Firstly, an acute preliminary experiment, where INCO group’s subjects inhaled a mixture of CO in O_2_ (4 L) over a 2 min time period by mask (1 mL/kg of body weight). The individuals in the INCO group remained at rest after inhalation of CO. Exhaled air was analyzed in numerous time points including pre, immediately post, and at each 1, 2, 4, 6, and 8 h interval post CO inhalation for exhaled gas CO concentration. EPO concentration was measured in venous serum (15 min, 3000 rpm) and venous blood HbCO% was measured at the same time points by Radiometer ABL 725.

#### 4-Week of Periodic CO Inhalation Testing

The second experimental section examined the effects of 4-week of periodic CO inhalation combined with exercise training on maximal aerobic power. Both pre- and post-training, the total hemoglobin (tHb), red blood cells (RBC), hematocrit (Hct), hemoglobin (Hb), mean corpuscular volume (MCV), running economy (RE), and VO_2_max were measured and compared between two groups, respectively. The exercise-training program consisted of treadmill running for 4-weeks, 5 times a week, and 50 min per session. Prior to each training session, all subjects inhaled gas for a period of 2 min continually experiment group (INCO): A mixture of CO (1 mL/kg of body weight) in O_2_ (4 L); Control group (NOCO): only O_2_ (4 L). CO inhalation occurred 15 min before commence of the training session. The treadmill speed commenced at 90% of ventilator threshold and was maintained for 40 min. 5 min prior to and post each run, subjects completed a warm up and cool down, resulting in a total running time of 50 min. At the end of each training session, the participants were requested to provide ratings of perceived exertion (RPE) scores. When the RPE decreased two or more units, the speed of the next training session was increased by 5%.

### Determination of the RBC, Hct, Hb, and MCV

Blood red blood cells (RBC), hematocrit (Hct), hemoglobin (Hb), and mean corpuscular volume (MCV) tested from the venous blood samples by Bayer ADVIA 120, Germany.

### Determination of Maximum Oxygen Uptake (VO_2_max)

VO_2_max was measured twice, once 24 h prior and again 24 h post 4 weeks of training, utilizing an incremental running test (Treadmill: h/p/cosmos, CORTEX Metalyzer 3B, Germany). The protocol included an initial 3-min warm up at 5 km/h and the speed was increased to 9 km/h. Treadmill speed was then increased by 1 km/h every minute, until the subjects were unable to continue or had reached the VO_2_max plateau. Subjects then cooled down at 5 km/h for 5 min.

### Determination of Running Economy

Running economy was measured twice, before and after 4 weeks of training. The testing equipment was the same as with VO_2_max test. The protocol included an initial 3-min warm up at 5 km/h, and the speed was increased to 8 km/h and lasted for 8 min until steady O_2_ uptake had been reached. Then the speed was increased to 10 and 12 km/h individually for testing the running economy and these stages also lasted for 8 min.

### Determination of Exhaled Gas and Blood CO Concentration

In the preliminary experiment, the concentrations of exhaled CO and venous blood (HbCO%) were measured pre and post CO inhalation (pre, 0, 1, 2, 4, 6, and 8 h). The CO concentration in exhaled (end-tidal) gas was measured using the Drager Pac 7000 CO (Germany) and HbCO% in blood was analyzed via Radiometer ABL 725 (Radio, United States).

### Measurement of EPO

5 mL blood samples were drawn from cubital vein. Serum EPO was measured via ELISA (ELISA kit: Human EPO ELISA Kit, Sigma, United States; xMARK, Bio-rad, United States). The sensitivity of the test was 0.16 mIU/mL. The coefficient variable of intra-assay was 4.0% and the CV of inter-assay was 7.3%.

### Determination of Hemoglobin Mass and Total Blood Volume

The measurements of tHb, total blood volume (BV), Plasma volume (PV), and Red cell volume (RCV) were determined through the CO-rebreathing method, according to the Durussel’s protocol (Kjellberg et al., 1949; Fröscher and Uhlmann, 2016). We measured these parameters twice, once 3 days prior to the start of training, and 3 days post the 4-week training intervention.

### Statistics

Values are reported as mean ± standard deviation. Statistical analyses were performed by an analysis of variance (ANOVA) for repeated measures (acute experiments) and pre- and post-training intervention (2-way ANOVA). When significant F-ratios were detected, Student-Newman–Keuls *post hoc* tests were used to identify differences between time points or the INCO and NOCO conditions. A difference of *P* < 0.05 was classified as significant, and a difference of *P* < 0.01 was classified as very significant. The magnitude of changes was expressed as standardized mean differences (Effect Size, ES) (Cohen, 1988). ES-values between 0.2 and 0.49 indicated a small difference, between 0.5 and 0.79 as a medium difference, and from 0.8 and above as a large difference. All data analyses were carried out through SPSS 23.0.

## Results

### Preliminary Experiment

**FIGURE 2:**
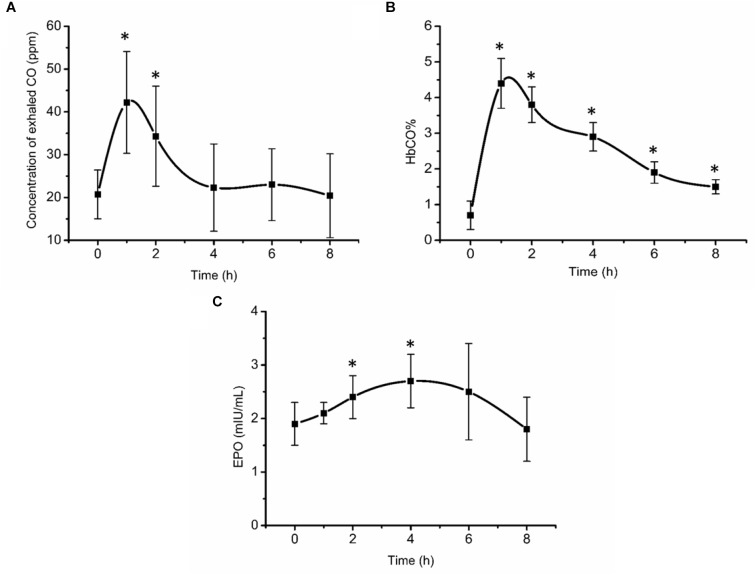
Exhaled CO concentration **(A)**, blood HbCO% **(B)**, and EPO concentration **(C)** after acute inhalation of CO. ^∗^Compared to baseline value *P* < 0.05. The HbCO% and EPO are percentage of carboxy-hemoglobin and erythropoietin hormone, respectively.

Exhaled CO concentration, blood HbCO% and EPO concentration initially increased after inhalation of CO (Figure 2). 1 and 2 h post inhalation, CO concentrations in exhaled gas were elevated (*P* < 0.05), returning to baseline 4 h post inhalation (Figure 2A). After initial increase, blood HbCO% (Figure 2B) fell with a clearance half-time of 5 h, but was still elevated 8 h post inhalation (*P* < 0.05). Serum EPO concentration (Figure 2C) increased significantly 2 h post inhalation (*P* < 0.05), peaking at 4 h post inhalation at 2.7 mIU/mL (an increase of 42.1% from baseline values), then gradually decreased over time.

### Exercise Training and CO Inhalation

Blood parameters in the INCO group and the NOCO group were measured 3 days pre and post of the 4-week training intervention as presented in Table 2. Post-training, a significant (*P* < 0.05) increase was observed in tHb (3.7%, ES = 0.76), RCV (5.3%, ES = 2.14), PV (6.8%, ES = 6.34), and BV (6.2%, ES = 39.45) in the INCO group. In contrast, NOCO group had no significant differences post-training intervention. Further, the hematological parameters of pre- and post-training intervention are shown in Table 3, and the values of these variables (RBC, Hct, Hb, and MCV) were not significantly different between the two conditions.

**Table 2:** Total blood parameters before and after 4 weeks of training.

	INCO (*n* = 6)			NOCO (*n* = 6)		
	Pretest	Posttest	Difference (%)	Pretest	Posttest	Difference (%)
tHb (g)	919 ± 45	953 ± 26^∗#^	3.7 (ES = 0.76)	887 ± 27	912 ± 17	2.8 (ES = 0.93)
RCV (mL)	2882 ± 72	3036 ± 128^∗#^	5.3 (ES = 2.14)	2735 ± 83	2789 ± 83	2.0 (ES = 0.65)
PV (mL)	4073 ± 44	4352 ± 19^∗#^	6.8 (ES = 6.34)	3757 ± 220	3783 ± 171	0.7 (ES = 0.12)
BV (mL)	6954 ± 11	7388 ± 7^∗#^	6.2 (ES = 39.45)	6492 ± 141	6572 ± 131	−1.2 (ES = 0.57)

*^∗^Comparing INCO before and after training P < 0.05; ^#^Comparing INCO to NOCO after training P < 0.05. The tHb, RCV, PV and BV are total hemoglobin, erythrocyte volume, plasma volume and total blood volume, respectively. INCO, training group with CO inhalation; NOCO, control group with no CO inhalation.*

**Table 3:** Hematological parameters before and after 4 weeks of training.

	INCO (*n* = 6)			NOCO (*n* = 6)		
	Pretest	Posttest	ES	Pretest	Posttest	ES
RBC (10^9^/L)	5.0 ± 0.3	5.1 ± 0.2	0.33	5.1 ± 0.3	5.1 ± 0.3	0
Hct (%)	0.41 ± 0.02	0.42 ± 0.01	0.50	0.42 ± 0.02	0.42 ± 0.03	0
Hb (g/L)	132 ± 4	131 ± 6	0.25	136 ± 7	139 ± 34	0.43
MCV (fL)	86 ± 2.4	86 ± 2.2	0	86 ± 1.5	87 ± 0.9	0.67

*There were no differences between pretest and posttest in RBC, Hct, Hb, and MCV. The RBC, Hct, HGB, MCV, and ES are red blood cells, hematocrit, hemoglobin, mean corpuscular volume, and effect size, respectively. INCO, training group with CO inhalation; NOCO, control group with no CO inhalation.*

### Maximal Aerobic Power After 4 Weeks of Training

The absolute and relative values of VO_2_max in the INCO condition significantly increased by 2.4 and 2.7% (*P* < 0.05, ES = 0.33 and 0.70), respectively, post 4 weeks of training. The NOCO condition showed no significant differences (0.1%, ES = 0.014). The VO_2_max in the INCO group was significantly higher (*P* < 0.05) post-training when compared to the NOCO group. The data are presented in Table 4.

**Table 4:** VO_2_max before and after 4 weeks of training.

	INCO (*n* = 6)			NOCO (*n* = 6)		
	Pretest	Posttest	Difference (%)	Pretest	Posttest	Difference (%)
VO_2_max (L/min)	4.1 ± 0.3	4.2 ± 0.3^∗^	2.4 (ES = 0.33)	4.1 ± 0.3	4.1 ± 0.3	0.0 (ES = 0)
VO_2_max (mL/kg/min)	58.3 ± 2.3	59.9 ± 2.3^∗#^	2.7 (ES = 0.70)	58.4 ± 7.4	58.5 ± 7.7	0.1 (ES = 0.014)

*^∗^Comparing INCO before and after training P < 0.05; ^#^Comparing INCO to NOCO after training P < 0.05; INCO, training group with CO inhalation; NOCO, control group with no CO inhalation.*

At submaximal running speeds (8, 10, and 12 km/h), absolute/relative O_2_ uptake of both the INCO and NOCO group appears to be lower post-training, as shown in Table 5. However, the decrease was only significant in the INCO group (3.7, 3.6, and 5.4%, ES = 0.61, 0.48, and 0.63, respectively) (*P* < 0.05).

**Table 5:** Absolute/relative oxygen uptake at submaximal running speeds (L/min/mL/kg/min).

Running speed (km/h)	INCO (*n* = 6)			NOCO (*n* = 6)		
	Pretest	Posttest	Difference (%)	Pretest	Posttest	Difference (%)
8	3.02 ± 0.18/43.1 ± 2.6	2.91 ± 0.23/ 41.5 ± 3.3	−3.7^#^ (ES = 0.61)/ −3.7^#^ (ES = 0.62)	3.00 ± 0.22/ 42.2 ± 3.1	2.93 ± 0.15/ 41.3 ± 2.1	−2.3 (ES = 0.32)/ −2.1 (ES = 0.29)
10	3.30 ± 0.25/ 47.1 ± 3.6	3.18 ± 0.17/ 45.4 ± 2.4	−3.6^#^ (ES = 0.48)/ −3.7^#^ (ES = 0.47)	3.32 ± 0.12/ 46.8 ± 1.7	3.31 ± 0.26/ 46.6 ± 3.7	−0.3 (ES = 0.08)/ −0.4 (ES = 0.12)
12	3.51 ± 0.30/ 50.1 ± 4.3	3.32 ± 0.60/ 47.4 ± 8.6	−5.4^#^ (ES = 0.63)/ −5.4^#^ (ES = 0.63)	3.52 ± 0.07/ 49.6 ± 1.0	3.49 ± 0.23/ 49.2 ± 3.2	−0.9 (ES = 0.43)/ −0.9 (ES = 0.4)

*^#^Comparing INCO to NOCO after training P < 0.05. The ES means effect size. INCO, training group with CO inhalation; NOCO, control group with no CO inhalation.*

## Discussion

The results of the present study demonstrated that EPO concentration initially increased after one inhalation of CO (peaking at 4 h post inhalation (42.1%), then gradually decreased over time), also 4-week of sea level treadmill endurance training with an application of 1 mL/kg of CO inhalation 15 min prior to each 50-min training sessions increased subjects’ tHb (3.7%), plasma (6.8%), and whole blood volume (6.2%), VO_2_max (2.7%), and running economy (4%). Therefore, it is proposed that inhalation of CO is a practical method of inducing the performance enhancing physiological adaptations of hypoxia in well-trained athletes, without the logistical and financial burdens of traveling to altitude.

### Acute Effects of CO Inhalation on EPO Secretion

It is well-known that CO binds to Hb stronger than oxygen and is therefore potentially dangerous, especially when concentration is higher than 35 ppm or HbCO percentage of 15–20%. However, it is also widely used agent in medical research. CO re-breathing method is widely used to measure tHb, (Schmidt and Prommer, 2005; Jérôme et al., 2013) radiolabeled CO is used to measure regional tissue blood volume, (Heinonen et al., 2014) and CO is also used in physiological research to address fundamental aspects of cardiovascular regulation (González-Alonso et al., 2002). In the present study acute CO inhalation measures indicated that HbCO% was close to 5% at the highest (below 18 ppm) and gradually decreased to 2% at the 8 h time point. EPO is a glycoprotein secreted by cells in many tissues, mainly the renal cortical cells. When the O_2_ concentration in the renal cortex decreases, EPO secretion into the blood increases, causing myeloid progenitor stem cells to promote erythropoiesis (Sasaki, 2003; Lee and Percy, 2010). This occurs when low landers travel to altitude, due to the reduction in barometric pressure at altitude, which results in hypoxemia. Training at altitude takes advantage of this response, where hypoxia independent of training, stimulates EPO secretion (Schmidt et al., 1991; Volker, 2013). As a result, Hb increases and O_2_ delivery to the muscles is enhanced, enabling athletes to perform better on their return to sea level for competition (Geiser et al., 2001). In the preliminary experiments of the present study, approximately 2 h post CO inhalation, EPO concentration increased significantly by 26.9% and later by 42.3% at 4 h post CO inhalation. These results are similar to those previously documented by Eckardt et al. (1989) who determined that in subjects who were exposed to simulated altitude of 3000 and 4000 m, respectively, EPO significantly increased at 114 and 84 min, respectively, with a percentage increase of 40.6 and 67.7%, respectively. Therefore, it can be concluded that the current clinically safe dose of 1 mL/kg BW of CO as also previously utilized by Schmidt and Prommer (2010) is sufficient to induce a hypoxic state *in vivo* stimulating also EPO release.

It is known that CO binds avidly to Hb, with an affinity approximately 200-fold higher than that of O_2_ (Karen and Jean, 2007). When CO is inhaled, it readily displaces O_2_ from its binding sites on Hb. This occurs without a reduction in arterial PO_2_. While altitude results in reduction in both arterial PO_2_ and HbO_2_ concentration, inhalation of CO reduces only arterial HbO_2_ concentration. Therefore, with HbCO potentially impairing O_2_ delivery to cells, cellular hypoxia may develop in spite of a normal arterial PO_2_ as the plasma oxygen carrying capacity is very limited triggering an increased EPO secretion by the kidneys. It has been shown that increased ventilation during exercise may accelerate the removal of CO from the blood, (Zavorsky et al., 2012) but in the present study this was not determined, but should be addressed further in the future.

### Training Adaptations as a Result of Repetitive CO Inhalations

#### VO_2_max

The increase of VO_2_max through altitude training is thought to be the result of a combination of factors including better oxygen carrying capacity and enhanced O_2_ kinetics (Gore et al., 2001; Wehrlin et al., 2006; Kane et al., 2016). Importantly, it should be noted that altitude-induced hypoxemia is well known to stimulate compensatory hyperventilation, causing systemic alkalosis. This results in renal excretion of sodium and bicarbonate in an attempt to normalize pH. The excretion of sodium and bicarbonate increases urinary loss of water, which may result in possible dehydration and an early (EPO-unrelated) increase in Hb and Hct, from the reduction in PV. This may in turn act to impair cardiac output and O_2_ delivery and athletic performance. In contrast, CO does not stimulate hyperventilation, as there is no basis for dehydration after CO inhalation. As a consequence, BV may be better maintained using CO to simulate altitude in comparison to altitude exposure. Indeed, our results seem to reflect this expectation (Table 4). Further, as by 3 days post the 4-week training intervention tHb increased by 3.7% (ES = 0.76) in the INCO condition and by 2.8% (ES = 0.93) in the control group, effect was evident and indirectly demonstrated the function of EPO. It is suspected that some potential reasons for this may have been due to the elevated tHb and thus prolonged half-time of CO (Zavorsky et al., 2014) as well as an existing elevation in tHb, due to highly trained status of the sample (Kjellberg et al., 1949). While the present study cannot establish cause and effect, the percentage increases in BV were similar to those of the VO_2_max (Tables 2, 4).

In this study, the increase of both tHb and RCV correspond to the results of Wehrlin et al. (2006) who after 24 days of altitude training showed that Hb mass increased by 5.3% and RCV by 5%. In addition, Friedmann et al. (2005) observed that elite athletes who lived and trained for 3-week at an altitude of 2100–2300 m, showed on average a significant similar increase in tHb (6%). In line with Friedmann et al. (2005) no significant difference in Hct was apparent in this study despite of the increase in tHb, when comparing pre and post-training measurements. It is suggested that the increase in PV may be the reason. However, contradictory to our results utilizing CO inhalation, Wehrlin et al. (2006) found no significant change in PV and BV post-training at altitude. Two of the fundamental factors that directly impact maximal aerobic power are O_2_ transport through the cardiovascular system and O_2_ utilization (Saltin and Strange, 1992). In particular O_2_ transport can be influenced by tHb in the blood, as well as BV (Martino et al., 2002). Hence, high tHb and BV are essential to have a high maximal aerobic power (Green et al., 1999). It has been suggested that a 1 g increase in tHb can increase VO_2_max by approximately 4 mL/min (Schmidt and Prommer, 2010). Moreover, a higher BV, whether via an increase in PV or erythrocyte mass, causes an increase in stroke volume, cardiac output and thus an elevation in VO_2_max. A change in BV of 1.0 mL triggers a variation in VO_2_max by 0.7 mL/g/min (Eastwood et al., 2012). However, if tHb rises excessively there is the chance of blood viscosity increasing and having a negative impact on cardiac output, (Rhodes et al., 2009) nulling any aerobic fitness benefits. It is assumed that in this study the increase in PV likely prevented an increase in blood viscosity and thus the higher tHb mass did not interfere with cardiac output, resulting in improved maximal aerobic performance.

#### Running Economy

**FIGURE 3:**
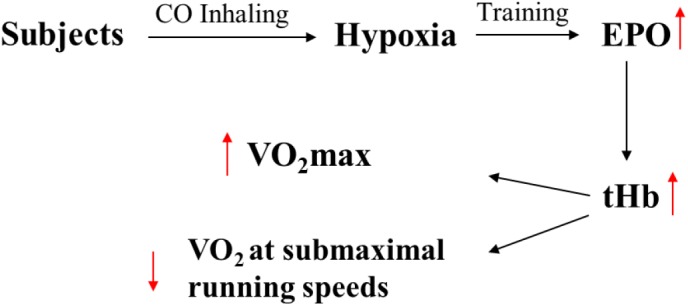
The assumed physiological reasons of improving in performance caused by inhalation of CO. The EPO and tHb are erythropoietin hormone and total hemoglobin.

Oxygen uptake marginally, but significantly decreased at submaximal speeds of 8, 10, and 12 km/h, suggesting an improvement in running economy (Figure 3). In accordance, research in hypobaric hypoxia with elite athletes, at submaximal intensities, has demonstrated that altitude training can enhance energetics through stimulating erythropoiesis, causing an increase in VO_2_max and a reduced energy cost of exercise (Daniel et al., 1986). A major contributories in the present study are thought to be CO’s ability to stimulate mitochondrial biogenesis, (Czuba et al., 2014) which can be partially initiated when CO binds to cytochrome c oxidase and heightens mitochondrial hydrogen peroxide formation (Suliman et al., 2007). Furthermore, CO acts similarly as nitric oxide, which has been shown to reduce skeletal muscle oxygen consumption in humans (Heinonen et al., 2011), which likely translates to benefit also exercising oxygen economy. Additionally, it has also been identified that post CO exposure, major histocompatibility complex class I (MHCI) protein expression is elevated in skeletal muscle, indicating a shift to efficient muscular phenotypes (Czuba et al., 2014). Finally, CO exposure may have an essential role in the preservation of cellular homeostasis at sites of muscle damage through the stabilization of HIF-1α, causing an up-regulation of TGF-α, leading to its pleiotropic effects (Chin et al., 2007). However, due to the premature and novel nature of this research, such mechanistic underpinnings can only be speculated. Nevertheless, it is probable that CO, *in vivo*, may have a complex multi-disciplinary contribution to improve running economy. Thus, further research is required to determine CO’s mechanistic role *in vivo* during exercise in the improvement of oxygen utilization economy.

### Limitations

This study included only fairly small number of subjects who all were football players. Therefore, these findings should be extended by studying larger amounts of subjects including also other sport disciplines. Furthermore, as excessive CO breathing can be detrimental and can even lead to death, CO breathing interventions such as presented here should only be done in well-controlled laboratory setting, where both exhaled and blood CO values are always closely monitored and appropriate first aid facilities are readily accessible if needed.

## Conclusion

Inhalation of CO (1 mL/kg BW) in normoxia stimulates EPO secretion in well-trained male soccer players. Inhaling 1 mL/kg CO prior to training sessions (five times/week for 4 weeks) increases PV, BV, RBC, tHb, running economy, and VO_2_max post-training and may thus be an alternative method to living and/or training at altitude, although no direct comparison to altitude training was performed in the present study. Therefore, it is proposed that inhalation of CO is a practical method of inducing the performance enhancing physiological adaptations of hypoxia, without the logistical and financial burdens of traveling to attitude. Furthermore, CO inhalation may also avoid the dehydrating diuresis typical in altitude, which can impair performance. However, it is emphasized that further research is warranted in regards to the ethical concerns of CO inhalation on individual athletes and in competitive sports.

## Ethics Statement

This study was carried out in accordance with the recommendations of sport science ethics review of Beijing Sport University, Beijing Sport University Institutional Review Board (BSU IRB), with written informed consent from all subjects. All subjects gave written informed consent in accordance with the Declaration of Helsinki. The protocol was approved by the “BSU IRB.”

## Author Contributions

All authors listed have made a substantial, direct and intellectual contribution to the work, and approved it for publication.

## Conflict of Interest Statement

The authors declare that the research was conducted in the absence of any commercial or financial relationships that could be construed as a potential conflict of interest.
